# A trend towards a more intense adjuvant treatment of low-grade-gliomas in tertiary centers in Germany after RTOG 9802 – results from a multi-center survey

**DOI:** 10.1186/s12885-018-4825-4

**Published:** 2018-09-21

**Authors:** Christoph Straube, Kerstin A. Kessel, Friederike Schmidt-Graf, Sandro M. Krieg, Bernhard Meyer, Jens Gempt, Stephanie E. Combs

**Affiliations:** 1 Department of Radiation Oncology, Klinikum rechts der Isar, Technical University of Munich (TUM), Munich, Germany; 2 Department of Neurosurgery, Klinikum rechts der Isar, Technical University of Munich (TUM), Munich, Germany; 3 Institut für Innovative Radiotherapie (iRT), Department of Radiation Sciences (DRS), Helmholtz Zentrum München, Munich, Germany; 4 Department of Neurology, Klinikum rechts der Isar, Technical University of Munich (TUM), Munich, Germany; 5 Deutsches Konsortium Translationale Krebsforschung (DKTK), Partner Site Munich, Munich, Germany

## Abstract

**Background:**

The treatment recommendations for Low-grade Gliomas (LGG) underwent profound changes due to results from RTOG 9802 published in April 2016. This work aims to investigate whether the results from the trial were already incorporated into the treatment recommendations at German oncology centers before an update of the official guidelines.

**Methods:**

An online based questionnaire with questions covering all aspects of adjuvant treatments of LGGs was generated, including three cases with distinct clinical situations. We contacted all members of the neuro-oncologic working group (NOA) of the German Cancer Society (DKG) as well as all German-speaking members of the European Low-Grade Glioma Network via E-mail.

**Results:**

We collected 38 responses. All responders were at least specialists; they predominantly worked at tertiary hospitals with a high volume of LGGs treated annually (75% with more than 10 cases per year). All responders stated to consent treatment recommendation for LGGs within interdisciplinary oncologic boards. The treatment recommendations for LGGs changed profoundly between 2015 and 12/2016. There is a trend towards PCV-based multimodal treatments, especially for oligodendroglial LGGs, as well as a trend away from watchful-waiting-policies for astrocytic LGGs.

**Conclusion:**

Neurooncologists do adapt results from clinical trials quickly. None the less, there is still an immense heterogeneity within the treatment recommendations, predominantly for astrocytic LGGs. Well planned clinical trials and concise treatment recommendations are warranted; additionally, individual counseling of patients is essential.

## Background

Low-grade Gliomas (LGG) are a rarely occurring malignancy that makes up 5–10% of all primary brain malignancies. Depending on the molecular pattern, on risk factors and treatment regimens, the median survival times range between 3.2 years and > 15 years [1, 2].

Initially, the management of LGG was mostly based on clinical risk factors, such as the age older than 40 years, the size of the lesion larger than 6 cm or the presence of neurological defects [1, 3]. As the survival-time of low-risk LGGs is significantly longer as compared to high-risk LGGs, clinical trials have focused on high-risk LGGs [1]. This limits the available knowledge concerning the treatment of low-risk LGGs [2, 4]. Besides that, new evidence suggests that the inclusion of molecular markers, especially IDH1 mutation status as well as the presence of LOH 1p19q, play a role as prognostic as well as predictive biomarkers [4, 5].

The most effective regimen for high-risk LGGs consists of an adjuvant fractionated radiotherapy up to a total dose of 54 Gy followed by six cycles of PCV (Procarbazine, CCNU, Vincristine). This regimen results in an increase of the median overall survival (mOS) from 7.8 years to 13.3 years and of the median progression-free survival (mPFS) from 4.0 to 10.4 years. The mature results of this trial were published in April 2016 [2]. However, the official EANO guideline was updated only recently [6].

We conducted a survey to answer the question, whether these results would have a sudden impact on the treatment regimens in German-speaking countries, already before adoption within the treatment guidelines.

## Methods

### Study design

We generated a questionnaire consisting of 17 questions about the infrastructure of the institutions as well as nine questions each about three fictional medical cases. The survey included 16 multiple-choice (MC) questions allowing only one answer, four MC questions allowing multiple answers and six questions with free answers. 12 of the MC questions also allowed to give a free response in case of missing or not precisely matching options.

### Cases

All three medical cases were fictional cases that were constructed to focus on relevant clinical situations. The first case was a 52-year-old male with a WHO°II Oligodendroglioma (IDHmt, LOH 1p19q, mMGMT) measuring 7 cm before surgery. The “patient” underwent subtotal resection (STR) and was in an excellent physical status (Karnofsky performance scale (KPS) 90%). This case was constructed as a standard situation for a high-risk Oligodendroglioma [2].

The second case was a 41-years-old female with a WHO°II diffuse Astrocytoma (IDHmt, no LOH 1p19q, mMGMT) that underwent gross total resection (GTR) and also was in an excellent physical status (KPS 90%). This case represents a high-risk Astrocytoma situation based on the inclusion criteria of the RTOG 9802 trial [2].

The third case was a 31-year-old female with a WHO°II diffuse Astrocytoma without IDH1 mutation, without LOH 1p19q, and without MGMT promoter hypermethylation. The patient underwent GTR and was in an acceptable physical Status (KPS 80%), yet there was some minor hemiparesis present after surgery. According to the RTOG 9802 inclusion criteria, this is a low-risk-case [2]. However, the molecular pattern of the tumor reflects a high-risk situation with a prognosis that is closer to Anaplastic Astrocytoma or even Glioblastoma [4, 6, 7].

### Questionnaire

The questionnaire was piloted by members of the departments of radiation oncology, neurology, and neurosurgery and reviewed by all authors for understandability. An ethical vote was not necessary, as there were no clinical data included and the survey is a pattern of care analysis.

The survey was generated as an online-based questionnaire at survio.com and invitations for the survey were send by e-mail to all 326 Members of the “Neuroonkologische Arbeitsgruppe” (neurooncological working group, NOA) of the “Deutsche Krebsgesellschaft” (German Cancer Society, DKG) as well as to all German Speaking Members of the European Low Grade Glioma Network (22 persons). The survey was open from December 12th, 2016 to January 30th, 2017.

## Results

### Responses and structural background

**Fig. 1:**
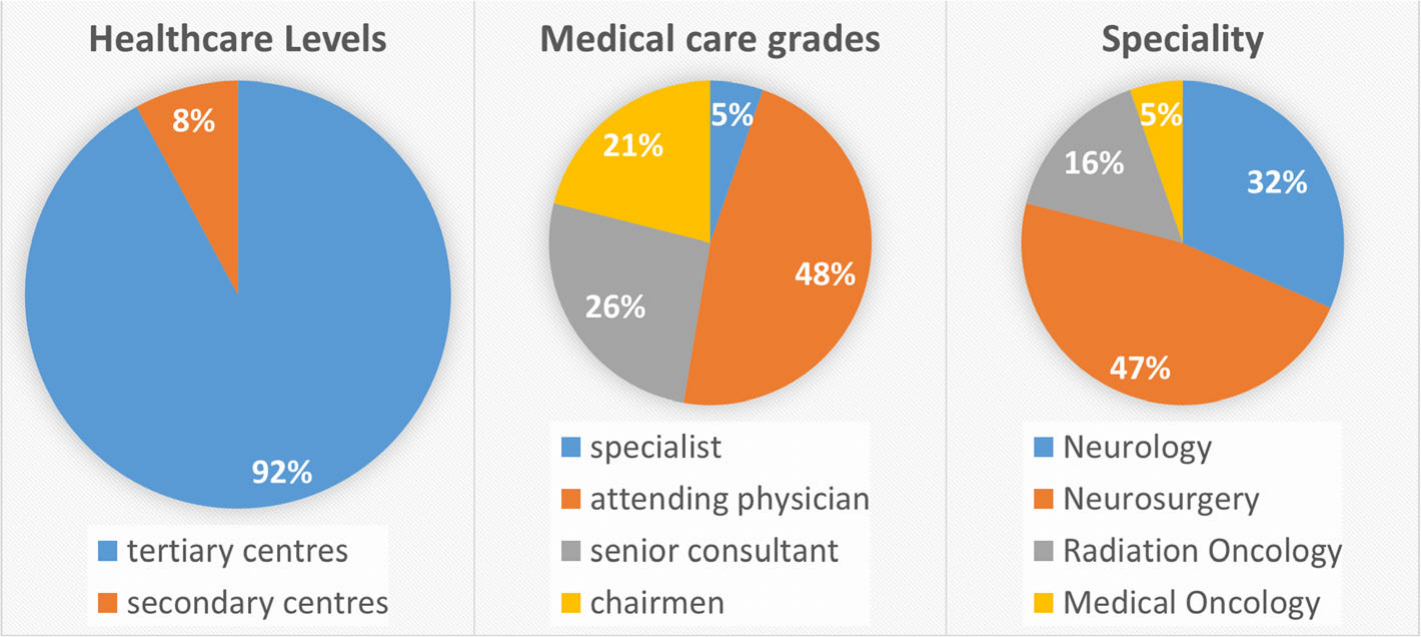
Composition of the responding cohort. Responders predominantly worked at tertiary centers (left panel), was in supervising positions (middle panel) and were predominantly neurosurgeons (right panel)

We counted 150 visits resulting in 38 completed surveys. 35/38 responders worked at tertiary care hospitals, and the remaining three responders worked at major regional hospitals (Fig. 1, left panel). Most responders worked in high-volume centers with > 10 LGG cases per year (15/38; 39,5%), > 20 LGG cases per year (9/38, 23.7%) or > 30 LGG-Cases per year (4/38, 10.5%). These numbers were educated guesses in 23 cases (60.5%) and numbers from a database in 12 cases (31.6%). The departments employed 23.5 physicians (median). Only specialists in their field answered the questionnaire, all (100%) of them answered that interdisciplinary oncologic boards provide treatment recommendations at their centers. 2/38 (5.3%) were specialists, 18/38 (47.4%) attending physicians, 10/38 (26.3%) senior consultants and 8/38 (21.1%) chairmen (Fig. 1, middle panel).

The majority of the responders were neurosurgeons (18/38, 47.4%), followed by neurologists (12/38, 31.6%), radiation oncologist (6/38, 15.8%) and medical oncologists (2/38, 5.3%) (Fig. 1, right panel).

The dataset is not representative of the members of the NOA. However, e-mail-communications by several of the responders suggest that in the majority of centers, only one person per center responded to the survey.

We asked for the technical abilities of the centers concerning imaging and radiation therapy. MRI with advanced sequences (Diffusion Weighted Images (DWI), Perfusion Imaging, Diffusion Tensor Imaging, etc.) was available at all centers. Positron Emission Tomography combined with computed tomography (PET-CT) or MRI (PET-MRI) were available at 86.8% and 21.1%, respectively. 25 responders gave answers for radiation oncology devices, one person did not answer this question at all, and 12 patients responded not to be able to answer this question. The majority of the remaining responders reported about providing advanced radiation oncology techniques, comprising 22/25 (88%) with intensity modulated radiotherapy (IMRT), 12/25 (48%) with image-guided radiotherapy (IGRT), 13/25 (52%) with frame-guided stereotaxia, 17/25 (68%) with frameless stereotaxia, 5/25 (20%) with MRI-Linear accelerators and 4/25 (16%) with particle therapy.

### Follow up-management

We then asked, which imaging as well as which radiation techniques are used for the target definition and treatment of LGGs. 13/38 responders stated not to be able to answer the question about the imaging used for treatment planning. From the remaining 25, MRI was used in all cases, PET-CT in 6/25 (24%), PET-MRI in two cases and SPECT in 1 case. Advanced MRI techniques, such as spectroscopy (3/25, 12%) and advanced sequences, such as DWI or Perfusion images were used by 8/25 (32%). 23 responders answered the question about the radiation techniques used for LGG patients. The majority used at least 3D conformal radiotherapy (20/23; 87%), 13 (57%) used IMRT or 3D-conformal techniques, and 2 (9%) used at least IMRT. One center stated to apply only frameless stereotaxia for the treatment of LGG patients. Particles were available in the centers of 4 responders; however, patients were either treated with photons or with particles in these centers. The centers mostly prescribed a dose of 54 Gy (median, range 50,4–60 Gy) in single doses of 2,0 Gy (median, range 1,7–2,7 Gy) centers.

The responders follow up their patient by MRI every three months (median, range 3–6 months) for two years (median, range 1–5 years), after that the imaging interval is prolonged.

### Cases

**Fig. 2:**
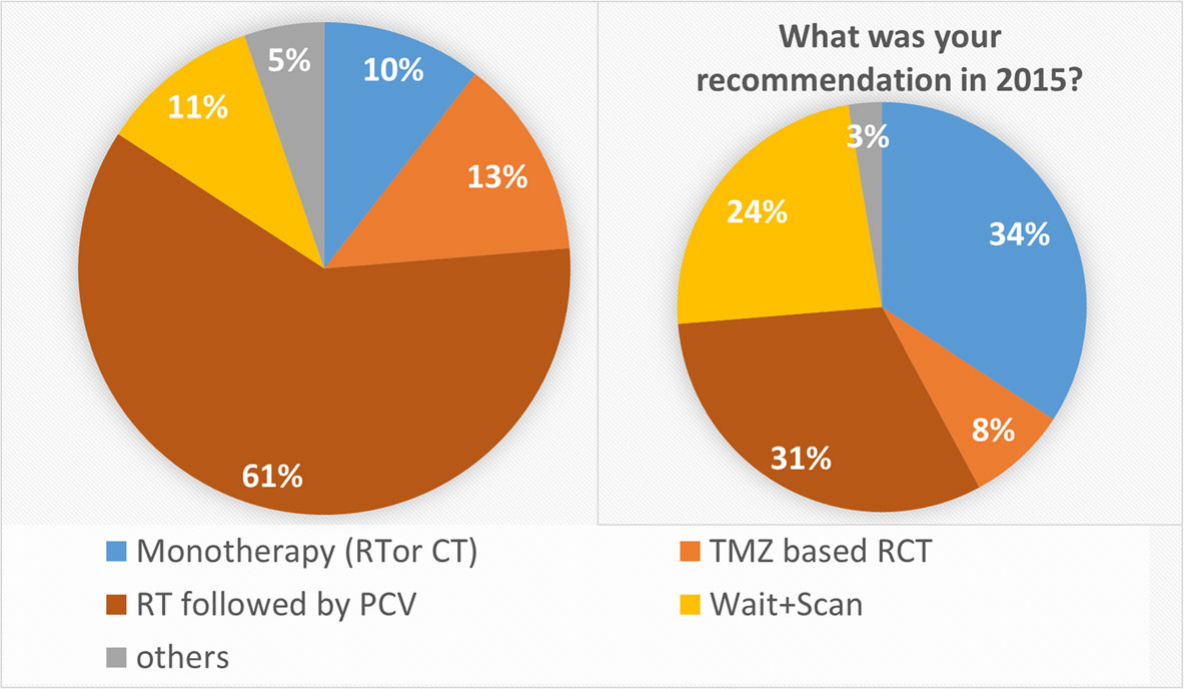
Treatment recommendations for case 1, a high-risk oligodendroglial LGG, in 12/2016 (left panel) and 2015 (right panel). After publication of the final results from RTOG 9802 and in comparison to 2015, patients with oligodendroglial LGGs were more likely to receive active treatments (89% vs. 76%), predominantly with radiotherapy followed by chemotherapy with PCV (61% vs. 31%)

All responders answered the questions to the 3 cases. In case 1 (Fig. 2), 61% would recommend radiotherapy followed by six cycles of PCV (RTOG 9802 regimen), 13% a radiochemotherapy with concomitant and adjuvant Temozolomide (TMZ-RCT). 10% would recommend monotherapy with either chemotherapy or temozolomide (TMZ). Further 11% would recommend a wait-and-scan policy. Asked for the treatment recommendation that would have been given in 2015 (i.e., before publication of the final results from RTOG 9802), only 31% would have recommended an RTOG 9802 regimen, but the majority of participants would have supported a mono-therapy with either chemotherapy or radiotherapy. A wait-and-scan policy was prescribed in 24% in 2015. In total, 14/18 (37%) reported that their treatment regimen was different in 2015 as compared to the end of 2016.

**Fig. 3:**
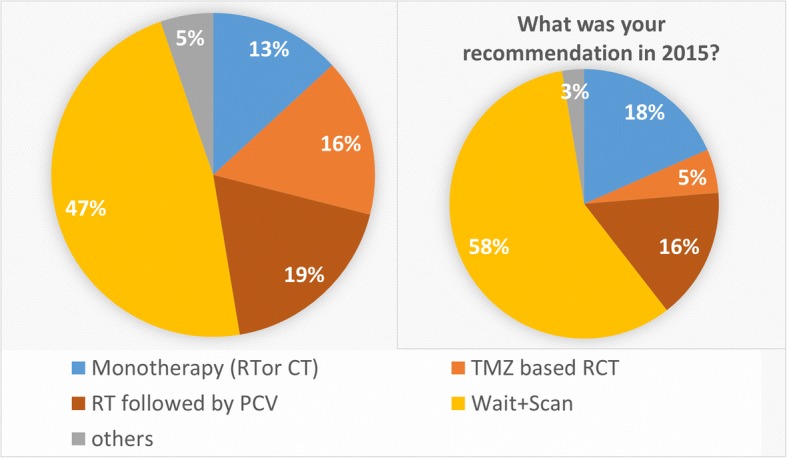
Treatment recommendations for case 2, a high-risk astrocytic LGG, in 12/2016 (left panel) and 2015 (right panel). When comparing the treatment recommendations before and after the publication of RTOG 9802, we saw a trend towards an active treatment also for high –risk astrocytic LGGs (53 vs. 42%). When patients received a recommendation for active treatment, this was most likely to be radiotherapy followed by PCV (19% in 2016 vs. 16% in 2015) or a radiochemotherapy with temozolomide (5% in 2015 vs. 16% in 2016)

In case 2 (Fig. 3), the recommended treatment in 2016 was wait-and-scan by 41% as compared to 58% in 2015. The RTOG 9802 regimen was recommended in 19% in 2016 as compared to 16% in 2015. A TMZ-RCT was recommended in 16% in 2016 as compared to 5% in 2015. Monotherapies were recommended in 13% in 2016 as compared to 18% in 2015. The recommendation differed from 2015 in 7/38 participants (18%).

**Fig. 4:**
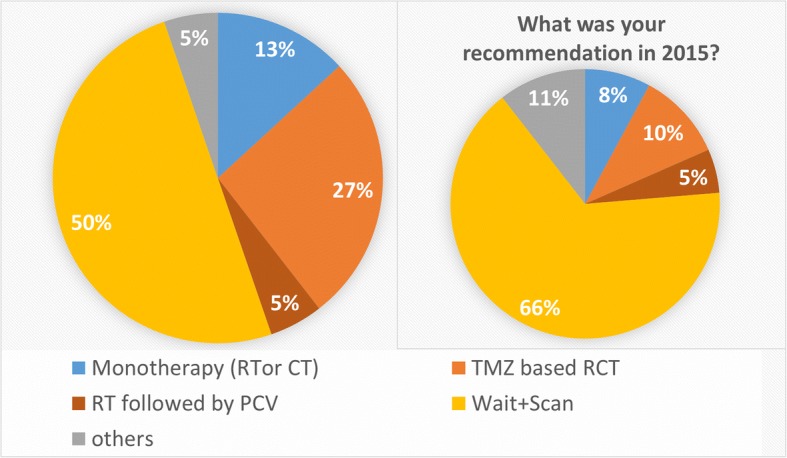
Treatment recommendations for case 3, a clinically low-risk LGG with a high-risk molecular pattern, in 12/2016 (left panel) and 2015 (right panel). Patients with clinically low-risk constellations but a high-risk molecular pattern were also more likely to receive active treatment after RTOG 9802 (34% in 2015 vs. 50% in 2016). Currently, a temozolomide-based radiochemotherapy is the preferred regimen within the centers (27% in 2016 vs. 10% in 2015)

Case 3 (Fig. 4) was a low-risk LGG by the extent of resection and the age of the patient but had a high-risk molecular pattern. In 2016 50% would have recommended a wait and scan policy, 27% a TMZ-RCT, 5% an RTOG 9802 regimen and 13% a mono-therapy. In comparison to this, the responders stated that the recommendation in 2015 would have been wait-and-scan in 66%, TMZ-RCT in 10% and an RTOG 9802 regimen in 5% of cases. 11% gave inconclusive answers, and 8% would have recommended a monotherapy in 2015. 8/38 (21%) participants reported that the recommendation in a similar case in 2015 would have been different.

## Discussion

### Neurooncologists adapt new results before their inclusion into guidelines

We have conducted a multi-institutional and multidisciplinary survey about the treatment of LGGs in German-speaking countries. Although the group of responders is not representative for all NOA members, the fact that all participants stated to consent treatment decisions within interdisciplinary boards allows an extrapolation towards the pattern of care in large neuro-oncologic centers in German-speaking countries.

It is important to reconsider the narrow timeline between the publication of the long-term results from the RTOG 9802 trial and our survey. While the manuscript was published on April 7th in 2016 [2], the survey was open from December 12th 2016 to January 30th in 2017. Of course, earlier results from the RTOG 9802 trial were available already in July 2012, but these findings did not show a significant survival benefit for the entire population yet [8]. As some centers already adapted the regimen before the long-term results were published in April 2016, the described trend, which was detected as short as 7 to 8 months after the publication of the long-term-results [2], can be interpreted as a general trend towards a more indents treatment of high-risk LGGs which was probably pronounced by the results from the RTOG 9802 trial.

According to the survey the centers adopted new results from large randomized trials already before new guidelines were published. This underlines the importance of multidisciplinary boards as well as of early communication of these results.

### Increase in RTOG 9802 regimens

Surprisingly, the results from the RTOG 9802 and the EORTC 22033 trials, did not influence the treatment recommendations of all histological subtypes, namely oligodendrogliomas, IDHmut diffuse astrocytomas, and IDHwt diffuse astrocytomas, to the same extent.

The most significant change in the treatment paradigm was recognized in the treatment of diffuse oligodendrogliomas. Within only one year, likely driven by the publication of the impressive 5.5-year overall survival benefit within the entire cohort, the acceptance of the RTOG 9802 regimen almost doubled from 31 to 61% [2]. This change was mainly due to a loss of acceptance of the wait-and-scan policy (24 vs. 11%) and monotherapy regimens (34 vs. 10%).

This follows the recommendations of the recent EANO guidelines, which were published post-hoc of the survey [6]. It seems likely that the positive subgroup analysis of RTOG 9802 for oligodendrogliomas as well as the results from trials for anaplastic oligodendrogliomas have further permitted the adoption of the RTOG 9802 regimen for this specific histological subtype [2, 9, 10].

An argument in favor of this assumption is that there were an only little increase and little total use of the RTOG 9802 in either IDH wt or IDH mut diffuse °II astrocytomas. Notably, case 2 was designed to fit within the inclusion criteria of the RTOG 9802 trial which showed a significant OS benefit not only for astrocytomas but also for IDH mut gliomas [2]. As a caveat, the report did not distinguish between IDH mutated astrocytomas and oligodendrogliomas. As the letter ones are mostly IDH mutated, a selection bias in favor of oligodendrogliomas cannot be ruled out for this subgroup analysis.

### Raise of TMZ-based RCT

The recommendations of TMZ-based RCT increased from 2015 to 2016 in LGGs with astrocytic histology. In case 2, an IDH mut clinical high-risk LGG astrocytoma this change (5 vs. 16%) was mainly driven by a reduced likelihood of a wait-and-scan policy (56 vs. 47%) and by a decrease in monotherapies. In case 3, where a clinical low-risk constellation was chosen and combined with a high-risk molecular pattern (IDH wt, MGMT promotor not methylated), the recommendation of a combined TMZ RCT increased from 10 to 27%. A preferred prescription of TMZ was also shown in a survey in Canada from 2015 [11]. This is in contrast to a National Cancer Database Analysis from 2017 which showed the only limited use of mono-agent chemotherapies (MAC) in the context of multimodal treatments of LGGs in the US [12].

There is only limited evidence that substantiates recommendations of a TMZ-based RCT for LGGs. The equal efficacy of TMZ in comparison to RT in LGG was shown by the EORTC 22033 trial [4]. Notably, the trial was formally negative, as it was designed to show a difference in PFS in favor of TMZ, and reports relatively early results. As one important result, the trial also showed no difference in the quality of life of patients treated within the two arms [13]. In the context of multi-modal treatments, the single-arm phase II RTOG 0424 trial showed an increased three year and OS for patients treated with TMZ-based RCT in comparison to historical controls treated with RT only [1, 14, 15].

The NOA-04 trial, which enrolled only patients with anaplastic gliomas, randomized the patients between three uni-modal regimens: (I) TMZ, (II) PCV and (III) RT. While the trial was not able to show an advantage of one regimen over another for the entire cohort, it showed that only patients with LOH 1p19q benefited from PCV more than from TMZ, but the former regimen was found to be more toxic [16, 17]. Consequently, patients without a LOH 1p19q do not benefit from PCV, at least in anaplastic astrocytomas.

Taken together, there is indirect evidence as well as evidence from a single armed phase II trial that supports the use of TMZ RCT in patients with LGG. As this regimen is significantly less toxic as compared to PCV, this regimen might be associated with increased tolerability especially in less fit patients.

### Watchful waiting

A substantial proportion of the responders recommended a wait and scan policy for patients with LGG after surgery. This was also shown in a survey of Canadian neurosurgeons [11]. As there was no difference in OS in EORTC 22845, which compared early vs. delayed RT, a wait-and-scan policy seems to be feasible [18]. However, the RTOG 9802 explicitly was positive for high-risk LGGs with IDH mutations (case 2) and did not show a long-term neurocognitive sequel [2]. Furthermore, the authors stated that the impressive survival benefit was not explainable by differences in the frequency of salvage therapies. This is an argument in favor of an early treatment strategy [2].

Noteworthy, patients with diffuse astrocytomas without IDH mutations are at substantial risk of a worse course of the disease [7], which might be interpreted as an argument in favor of an HGG-like treatment, even in the absence of high-risk clinical features [6].

While we did not ask for the reasons for choosing this regimen, we do believe that fear of neurocognitive side effects of RT might play a role. Neurocognitive deficits, indeed, have been associated with RT, yet there is a long delay between the treatment and the onset of symptoms [19]. Especially this argument has to be weighted with the OS benefit that potentially can be gained with the use of RCT, also in astrocytic LGGs [2].

## Conclusion

The interdisciplinary treatment of LGGs remains controversial. While diffuse WHO °II oligodendrogliomas are currently treated with a multimodal PCV-based regimen in the majority of centers, the treatment of astrocytic LGGs is more heterogeneous. Especially for these cases, more concise treatment recommendations based on well planned prospective trials are warranted.
